# More than just a pair of blue genes: how cyanobacteria adapt to changes in their light environment

**DOI:** 10.1111/ppl.13178

**Published:** 2020-08-23

**Authors:** Robert H. Calderon

**Affiliations:** ^1^ Department of Plant Physiology Umeå University Umeå 90736 Sweden

## Abstract

Cyanobacteria require light to perform photosynthesis, but not all colors of light are equally useable for them. In particular, blue light‐grown cyanobacterial strains, including the well‐studied model organism *Synechocystis sp*. PCC 6803 (*Synechocystis*), have been observed to exhibit slower growth rates than white or red light‐grown cells. In this issue of Physiologia Plantarum, Luimstra et al. (2020) have attempted to understand why cyanobacterial cells suffer under blue light. They measured the molecular and genetic responses of *Synechocystis* cells to being shifted from white light to blue light. They found that blue light‐grown cells make changes that lead to a redistribution of energy flow between the two photosystems that power photosynthesis. These findings could help researchers identify avenues for optimizing photosynthesis in cyanobacterial species, a group of organisms which show great promise as potential solar‐powered factories for the production of biofuels and other high‐value products.

Human beings have a relationship with cyanobacteria not unlike our relationship with sharks. While we can sometimes see Spirulina being sold as a nutritional supplement or a friendly (and vegetarian) Great White Shark in animated films, humans generally regard sharks and cyanobacteria as dangers to human health. That sharks have sharp teeth or that some cyanobacteria cause toxic algal blooms is indisputable, but these two groups also fill important ecological roles that deserve our appreciation. Cyanobacteria are the closest living relatives of the microbes responsible for oxygenating our atmosphere over 2 billion years ago and widely distributed on the planet to this day. They use photosynthesis to harvest sunlight, fix carbon dioxide and produce oxygen. For this reason, they are of great interest as potential solar‐powered producers of a wide array of products ranging from biofuels to cosmetics and more (Abed et al. 2009).

For cyanobacteria to conduct photosynthesis efficiently, they must closely coordinate the activities of photosystem II (PSII) and photosystem I (PSI). If one of the two photosystems become damaged or its activity slows down, the activity of the other photosystem must adjust accordingly. Not doing so could lead to imbalances in the cell that reduce its chances of growing or even surviving. One of the factors that can lead to an imbalance between the two photosystems is the quality (color) of light. Specifically, blue light has been hypothesized to cause overexcitation of PSI at the expense of PSII (Luimstra et al. 2018).

In this month's issue of Physiologia Plantarum, Luimstra et al. have looked at the transcriptional response of *Synechocystis sp*. PCC 6803 (hereafter *Synechocystis*) after exposure to blue light. It has been previously observed that cyanobacteria have lower growth rates under blue light than under standard white light conditions (Wilde et al. 1997). Because blue light is disproportionately used by PSI at the expense of PSII, Luimstra et al. hypothesized that cells would compensate for this by adjusting the PSI:PSII ratio in response to blue light.

The authors examined two possible ways that *Synechocystis* might adjust its PSI:PSII ratio, but first they confirmed that blue light‐grown *Synechocystis* do grow at a slower rate than red or orange light‐grown cells (Fig. 1A). They then looked at whether or not *Synechocystis* redistributes captured light energy between the two photosystems. Most cyanobacterial species use specialized light‐harvesting antennae called phycobilisomes (PCBs) that trap light energy and funnel it to both PSI and PSII (Fig. 1B). In response to blue light, the PCBs in *Synechocystis* cells remain attached to PSII but detach from PSI (Fig. 1C). This redistributes incoming light energy to PSII at the expense of PSI.

**Fig 1:**
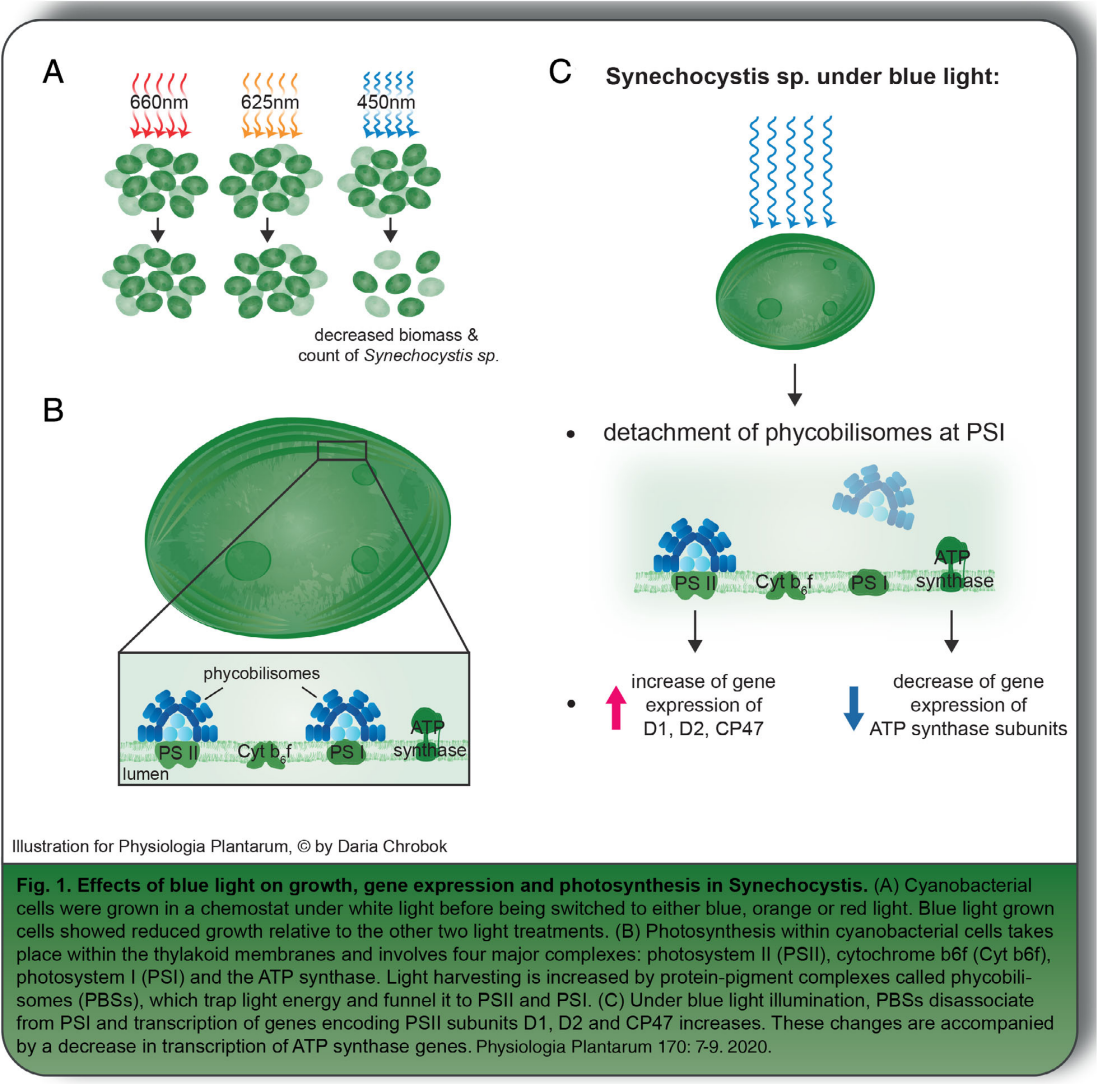


At the same time, they found that Synechocystis cells were specifically increasing the transcript levels of genes encoding subunits of PSII (Fig. 1C). These changes were detectable 4 h after exposure to blue light and persisted even 8 days later. The cells also appeared to be preparing for a reduction in photosynthesis by decreasing transcript levels of genes associated with ATP production, carbon fixation and protein translation. Interestingly, they massively increased the transcription of a set of genes associated with stress.

So, what is the implication of the work by Luimstra and colleagues? For starters, it lends further support to the hypothesis that blue light is preferentially absorbed by PSI over PSII in cyanobacteria. This can be seen in the detachment of light‐harvesting antennae from PSI and the increased transcription of PSII genes.

On a larger scale, their work also highlights one of the potential challenges in using bioengineered cyanobacterial strains to make high‐value products for humans. Efforts have already been made to expand the spectrum of light that cyanobacteria can use for photosynthesis. This includes bioengineering a strain of cyanobacteria to be able to absorb and use far‐red light for photosynthesis (Ho et al. 2016). There have been some attempts at improving the usage of blue light for photosynthesis in cyanobacteria. These approaches have focused on expressing plant light‐harvesting proteins in cyanobacteria, a method that has unfortunately not yet been successful. This is likely due to the difficulty of ensuring proper folding of these proteins in a different organism (Jensen and Leister 2014). Because the results of Luimstra and co‐workers have focused on the native response of cyanobacteria to blue light, their work could lead to new approaches for better using blue light that bypass this problem altogether. Manipulating the expression of some of the genes identified in this study, for example, could lead to strains of cyanobacteria that are better suited for the artificial growth environments required for the production of biofuels. Perhaps the appreciation for these biofuel‐producing strains will finally lead to cyanobacteria getting the respect that they deserve.
